# Advancement in sequencing the mitochondrial genome of *Birmella discoidalisa* Wei, 1994 (Hymenoptera: Tenthredinidae) and the phylogenetic classification of Fenusini

**DOI:** 10.1080/23802359.2019.1692728

**Published:** 2019-11-20

**Authors:** Ruoxuan Wu, Meicai Wei, Mengmeng Liu, Gengyun Niu

**Affiliations:** a College of Life Sciences, Jiangxi Normal University, Nanchang, Jiangxi, China;; b Shanghai Key Laboratory of Plant Molecular Sciences, College of Life Sciences, Shanghai Normal University, Shanghai, China

**Keywords:** Mitochondrial genome, next-generation sequencing, phylogeny, Tenthredinidae, *Birmella*

## Abstract

The nearly complete mitochondrial genome of *Birmella discoidalisa* Wei, 1994 has been sequenced and the genome was revised with more comprehensively sequenced to near completion. The new mitogenome sequences were constructed using two separate assembly approaches, both yielding consistent results. Compared with the sequence previously reported (MF197548.1), the *trnI* (*+*) and *trnQ* (*−*) genes were assembled, and the *trnI* (+)–*trnQ* (*−*) genes were rearranged compared with the ancestral type. The systematic classification of *B. discoidalisa* was examined to provide a basis for allocation into Tenthredinidae phylogeny.

Fenusini is a tribe of Tenthredinidae with a limited systematic characterization of its phylogenic position among sawflies. More than five different classification systems have been proposed for the systematic position of Fenusini (Benson 1952, 2009; Takeuchi 1952; Abe and Smith 1991; Wei and Nie 1998; Taeger et al. 2010). To clarify the systematic relationship of Fenusini with other branches of Tenthredinidae, it is necessary to sequence and analyze a mitochondrial genome of a species of the tribe.

*Birmella* is a common genus of Fenusini, occurring in southwestern China and Myanmar (Malaise 1964; Wei 1994). *Birmella discoidalisa* Wei, 1994 is endemic to southwestern China. In this study, the mitogenome of the tribe Fenusini was sequenced by next-generation sequencing. The mitogenome architectures and nucleotide compositions were briefly described.

**Figure 1.**
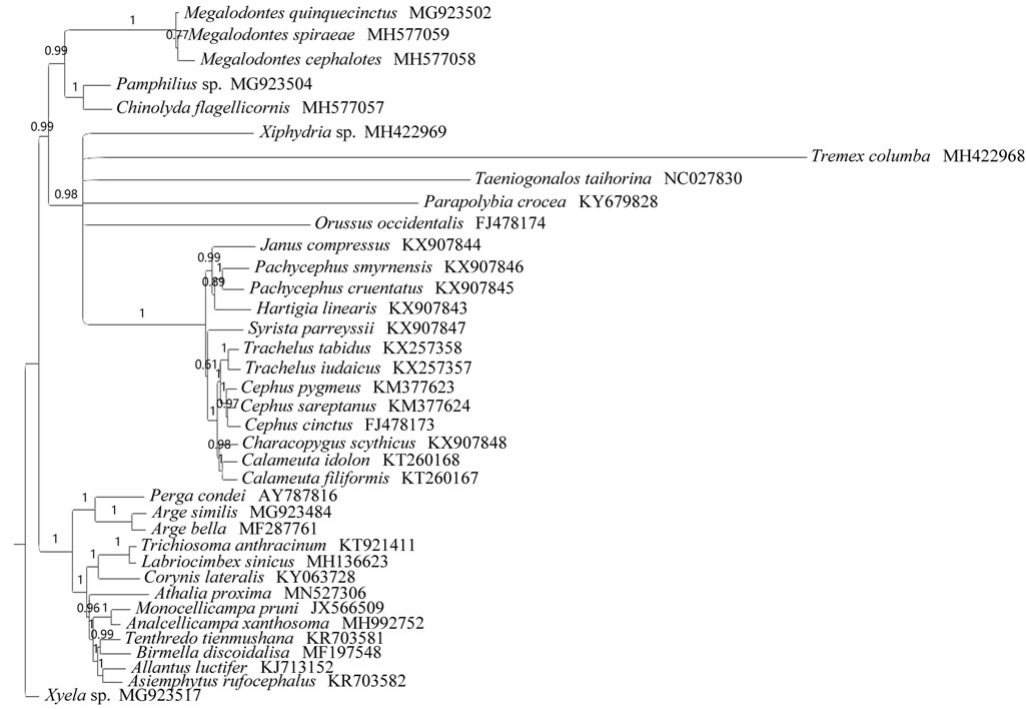
PhyloBayes tree based on the combined data of nine unsaturated protein coding genes and two RNA genes. Numbers above each node are posterior probabilities. The accession number of each species is indicated after the scientific name.

Samples of *B. discoidalisa* were collected from Leidongping, Sichuan, China (29.54°N 103.33°E) in May 2015. The specimens (CSCS-Hym-MC0029) from which the samples were taken are available at the Asia Sawfly Museum, Nanchang (ASMN) repository. Genomic DNA was sequenced using the high-throughput Illumina Hiseq 4000 platform, with 150 bp paired end reads. In total, 10,111,590 raw reads (SRR10429335) were obtained, and assembled using MitoZ (Meng et al. 2019) and Geneious Prime 2019.2.1 (https://www.geneious.com). *Kaliofenusa* sp. (undescribed new species from China) and *Fenusella taianensis* (Xiao et al. 1983) were used as references with the mean depth of coverage across the sequences being 1551 and 2496, respectively. All tRNAs were detected using the MITOS website (Bernt et al. 2013). The boundaries and locations of protein-coding genes (PCGs) and rRNA genes were manually determined by aligning with nearly 90 Symphytan mitogenomes.

The nearly complete mitochondrial genome of *B. discoidalisa* sequenced in this study was 15113 bp in length. This revised and more comprehensive version was deposited in GenBank with an accession number MF197548.2. The overall base composition was 42.4% A, 36.7% T, 12.4% C, and 8.5% G, with 79.2% AT. Compared with ancestral insect mitochondrial genome, only the *trnI* (+)–*trnQ* (−) of *B. discoidalisa* exchanged relative positions. Five PCGs begin with ATT start codon, while another five PCGs begin with ATG start codon. The rest use ATA as a start codon. All the PCGs terminate with stop codon TAA, whereas *nad3* ends with the incomplete codon T.

Nine unsaturated PCGs (*nad2*, *atp8*, *nad4L*, *nad6* were excluded) and two ribosomal RNA genes of 35 Symphytan and two Apocritan were subjected to Bayesian analysis with PhyloBayes under the CAT-MtArt model (Lartillot et al. 2009) conducted on the CIPRES webserver (Figure 1). All related files have been uploaded to figshare (https://figshare.com/account/home#/projects/70820). One consensus tree was obtained, with *B. discoidalisa* standing as the sister group of *Tenthredo*. Investigations into more species are required to better represent Fenusini. This will enhance solving the phylogeny of Tenthredinidae.
